# A Novel FDLSR-Based Technique for View-Independent Vehicle Make and Model Recognition

**DOI:** 10.3390/s23187920

**Published:** 2023-09-15

**Authors:** Sobia Hayee, Fawad Hussain, Muhammad Haroon Yousaf

**Affiliations:** 1 Department of Computer Engineering, University of Engineering & Technology, Taxila 47050, Pakistan; sobia_h@yahoo.com (S.H.); haroon.yousaf@uettaxila.edu.pk (M.H.Y.); 2 SWARM Robotics Lab, National Center of Robotics & Automation (NCRA), Taxila 47050, Pakistan

**Keywords:** VMMR, multiclass classification, ambiguity, multiplicity, hybrid CNN model, Fisher discriminative least squares regression, small-scale fine-grained vehicle datasets

## Abstract

Vehicle make and model recognition (VMMR) is an important aspect of intelligent transportation systems (ITS). In VMMR systems, surveillance cameras capture vehicle images for real-time vehicle detection and recognition. These captured images pose challenges, including shadows, reflections, changes in weather and illumination, occlusions, and perspective distortion. Another significant challenge in VMMR is the multiclass classification. This scenario has two main categories: (a) multiplicity and (b) ambiguity. Multiplicity concerns the issue of different forms among car models manufactured by the same company, while the ambiguity problem arises when multiple models from the same manufacturer have visually similar appearances or when vehicle models of different makes have visually comparable rear/front views. This paper introduces a novel and robust VMMR model that can address the above-mentioned issues with accuracy comparable to state-of-the-art methods. Our proposed hybrid CNN model selects the best descriptive fine-grained features with the help of Fisher Discriminative Least Squares Regression (FDLSR). These features are extracted from a deep CNN model fine-tuned on the fine-grained vehicle datasets Stanford-196 and BoxCars21k. Using ResNet-152 features, our proposed model outperformed the SVM and FC layers in accuracy by 0.5% and 4% on Stanford-196 and 0.4 and 1% on BoxCars21k, respectively. Moreover, this model is well-suited for small-scale fine-grained vehicle datasets.

## 1. Introduction

Intelligent transportation systems (ITS) are essential components of smart city initiatives in urban areas worldwide to achieve optimal, safe, and sustainable utilization of the available transportation infrastructure and maximum traffic efficiency. Automatic vehicle analysis is significant in any intelligent transportation system involving vehicle attribute recognition, such as vehicle re-identification, vehicle type recognition, and VMMR (vehicle make and model recognition). VMMR has many applications, such as in surveillance for policing and law enforcement, augmenting Automatic License Plate Recognition (ALPR) systems, advanced driver assistance systems (ADAS), electronic toll collection (ETC), self-driving cars, intelligent parking systems, measurement of traffic parameters like vehicle count, speed, and flow, as well as market analysis for car manufacturing companies. Traffic monitoring via VMMR is a critical tool for gathering statistics that aid in designing and planning sustainable and efficient transportation infrastructure.

VMMR is fraught with complications. The first is vehicle detection; the VMMR system should accurately locate vehicles in video images to perform feature extraction and classification. Numerous vehicle variations, such as color, size, and shape, make the problem challenging. Furthermore, under different lighting conditions and viewpoint variations, the visual properties of vehicles also change dramatically. The next task is to classify the localized image regions into make and model categories. Unfortunately, good classification accuracy can be achieved only after addressing several issues. Firstly, the wide range of makes and models seen in practice can render the number of classes considered rather large, making it a challenging fine-grained classification problem. Next, different models from the same manufacturer (make) frequently share similar shape characteristics and are thus difficult to distinguish. Additionally, the same model can have various facelifts released by the manufacturer over the years, introducing intra-class variation.

For a long time, the performance of computer vision techniques was the primary bottleneck for camera-based traffic monitoring systems. However, the advent of deep learning has fundamentally altered the situation. Researchers must meet several challenges for a wholly integrated AI-based traffic surveillance infrastructure [1]. One of these is accident prevention and vehicle re-identification (reID), which allows a vehicle’s route to be calculated for different areas thanks to its unique visual characteristics [2]. VMMR systems come into play in these scenarios, making it possible to detect a vehicle’s brand, model, and color from the image. Our proposed approach and a real-time vehicle detection system can address this challenge. Image classification, in particular, has advanced to an entirely new level over the last decade, approaching human-level accuracy in several domains. An essential factor in this transformation is the availability of large-scale datasets. This paper treats the vehicle make and model classification as a fine-grained image classification problem. We use preexisting convolutional neural network (CNN) models for feature extraction and replace the fully connected (FC) layer with a customized classifier based on Fisher discriminative least squares regression (FDLSR) [3]. Our proposed method yields better results than standard transfer learning techniques. The main contributions of our paper are:

- Our technique combines deep features with FDLSR and SVM [4] to yield better classification accuracy.
- We have suggested a robust and efficient view-independent car make and model classification technique.
- Our proposed classifier can be trained on deep fine-grained features at low computational cost and has a short runtime.
- We have applied our proposed classifier to a number of publicly available datasets. The results obtained are comparable to state-of-the-art techniques.

The rest of the paper is arranged in the following manner. Section 2 describes the technical details of the proposed classifier in detail. Section 3 discusses the datasets used for training and testing our classifier, explaining the methodology of our proposed solution to vehicle make and model recognition, and Section 4 reports experimental results on Stanford Cars [5] and a Pakistani on-road car dataset. Finally, Section 5 contains concluding remarks and discusses future research directions.

## 2. Related Work

Fine-grained image classification aims to classify subcategories of a larger category through fine-grained images [5]. As our goal is fine-grained vehicle classification, we must build a model to identify the most discriminating image features. Therefore, it is vital to detect subtle differences in similar regions. Different subcategories generally have very similar appearances, but the various subcategories are occasionally inconsistent. Many visual disturbances, such as light intensity, occlusion, and blur, seriously reduce the classification accuracy of vehicles.

Vehicle analysis starts with vehicle detection. Once the vehicle is detected, we can classify it based on its class (car, bus, truck), make (Toyota, Honda, Ford), color (white, black, red, gray), or make and model (VMMR). VMMR methods belong to three main categories of fine-grained recognition: attention mechanism [6], high-dimensional feature coding [7,8], and specific characteristics [9]. To detect the primary class of a vehicle, several basic geometric parameters, such as length, width, and height, are approximated [10,11]. Kafai [12] and Grimson [13] processed spatial and edge-based vehicle features with a Bayesian decision rule for classification. Kumar [14] detected vehicle logos using a Haar cascade classifier and trained an SVM classifier to classify vehicles into four categories. To classify vehicles, some researchers used adaptive background models [15], multiclass SVM-based models [16], and 3D vehicle features and models [17]. Zhang [18] proposed a modified form of the classified vector quantization (CVQ) approach for vehicle type recognition, rejecting low-confidence samples and achieving reliable classification results.

Vehicle type classification is also explored by using vehicle geographical features [19], edge-based features [20], histogram of gradient (HoG) features [21], contour point features [22], curvelet transform features [23], and contourlet transform features [24]. Some studies combined two features, such as wavelet and contourlet features, to improve results [25], as well as PHOG and Gabor features [26]. Dong et al. [27] achieved 83% to 98% accuracy. Liao et al. [9] proposed a strong-supervised DPM (SSDPM) for semantic segmentation of frontal vehicle images. Liao et al. used a novel symmetrical SURF descriptor to improve the discriminative powers of different parts, and the proposed method recognized the brand of each vehicle based on the weights of these parts. Hu and Psyllos [28] focused on brand recognition of a vehicle using discriminative pattern learning, car logo matching, and classification. Loua [29] implemented Lowe’s [30] approach of keypoint localization and SIFT features for make and model vehicle recognition. It matched features tie-breakly, but the algorithm proved ineffective in overall vehicle make and model recognition. In addition to SIFT, other features based on edges, gradients, or corners [31], and MPEG-7 descriptors such as edge histograms [32] were also explored for VMMR purposes. In [31], He et al. used Sobel and Canny edge detectors to detect texture, boundaries, and line segment maps of headlamps and license plates. SURF descriptors gained the attention of many researchers due to their fast processing. Siddiqui et al. [33] extracted SURF features from vehicles’ front or rear images and embedded them into a bag of sped-up robust features (BoSURF) histograms. Hsieh [34] used a grid division scheme and a combination of the histogram of gradient (HoG) and SURF descriptors to detect the region of interest and extract features from the vehicle. The low accuracy in [20] indicated that locally normalized Harris strengths (LNHS) were inefficient for the VMMR problem. However, the shape-based feature approaches, which extract features from vehicle backlights [35] and rear emblems [36], showed encouraging recognition rates in vehicle make and model recognition.

Model-based vehicle recognition uses the adaptive model [37], the approximate model [38], and the 3D model [39]. In [39], Prokaj and Medioni adopt the model-based approach and project the pose of a 3D CAD vehicle model to a 2D vehicle image to calculate the similarity score. Several classification approaches are proposed to improve VMMR classification. Psyllos et al. [40] classify SIFT features extracted from vehicle images using a probabilistic neural network. Pearce and Pears [20] investigate VMMR classification using the k-nearest neighbor classifier and the naive Bayes classifier. He et al. use neural networks and AdaBoost, SVM, and KNN for classification [31]. Random forest [41] and the nearest neighborhood classification approach [42] are also applied to identify the make and model of vehicles.

Fang et al. [43] proposed using CNNs to classify vehicles. SVM is also one of the popular classifiers in VMM classification [44]. A recent literature study shows that convolutional neural networks (CNNs) have set a new performance baseline in fine-grained visual classification [45,46,47,48,49]. Liu et al. [50] and Yang et al. [51] reinforced the viability of CNNs in fine-grained classification. Their work, GoogleNet, one of the first pre-trained deep learning models for fine-grained vehicle classification, outperformed the traditional approaches. Earlier research focused on auxiliary networks to learn local-level information for fine-grained classification. Krause et al. [52] proposed a fine-grained recognition method that worked without part annotations. They used the concept of alignment and segmentation to learn and detect useful parts. Xiao et al. [6] used three types of attention to extract relevant details of an image. They integrated these attentions to train deep nets. Zhang et al. [53] proposed an automatic fine-grained recognition approach, free of any object or part annotation. It extracted and pooled deep, distinctive filter responses and learned specific patterns significantly and consistently. Wang et al. [54] emphasized mid-level representations of CNNs, which collected the class-level discriminative information end-to-end. Zhang et al. [55] addressed the constraints in pose-normalized representations for fine-grained classification. They introduced semantic part localization in convolutional neural networks and achieved state-of-the-art results. Fu [56] proposed a recurrent attention model that learns discriminative region attention and region-based feature representation at multiple scales without using bounding boxes. A novel part-stacked CNN proposed in [57] encodes the object-level and part-level cues simultaneously to model the subtle differences between the object parts. Hu [58] introduced spatially weighted pooling (SWP) layers in CNN, which pools extracted features by learning the discriminative spatial units. The proposed method surpassed previous fine-grained vehicle classification methods. Ma [59] improved the generalization ability of a CNN model by inserting a channel max pooling (CMP) layer between convolutional layers and the fully connected layers. In lightweight convolutional neural networks (LWCNNs) [60], network parameters are minimized and optimized by pre-training, fine-tuning training, and transfer training on a VMMR dataset [51].

Lam et al. [61] defined a heuristic function that scored the proposals of informative image parts and unified them via a long short-term memory (LSTM) network into a new deep recurrent architecture. Lin et al. [62] proposed a valve linkage function (VLF) for back-propagation chaining, improving the fine-grained classification performance of deep localization, alignment, and classification (LAC) systems. Zhang et al. [63] introduced the semantic part detection and abstraction (SPDA) approach in mid-level layers of an end-to-end CNN model. This approach shares the computation of convolutional filters and achieves state-of-the-art results in fine-grained classification. Different entropy loss functions were introduced to improve the performance of end-to-end neural networks. Deep CNNs with large-margin softmax (L-softmax) loss [7] created desired margins among features, made them more discriminative, and provided better classification results. The center loss was designed by Wen et al. [8] to improve inter-class dispensation and intra-class compactness. It learned the center of each class and restricted the distance of deep features from their respective classes. Focal loss [64] improved the dense object detection results by addressing the class imbalance problem and proposed training of hard-set examples only. Lin et al. [64] proposed a new loss function, introducing a regularization term to cross-entropy (CE) loss, which penalized the probability of a data point being assigned to a class other than its ground-truth class. The back-propagation algorithm used in CNN training typically optimizes the loss function. In contrast, in fine-grained classification, general and redundant features are undesirable. Ma et al. [59] addressed this problem by inserting a channel max pool layer between the convolutional layers and the fully connected layers of the CNN. This layer aimed to improve the generalization ability of the CNN by learning more discriminative features from a relatively lower number of feature maps. Experimental results demonstrated that CNNs with a CMP layer improved the classification accuracies on fine-grained vehicle classification with massively reduced parameters. Chang et al. [65] proposed a single loss, mutual-channel loss (MC-loss), applied directly to the feature channels to obtain class-aligned discriminative and diverse features. Naseer [66] also reduced the feature space by applying the genetic algorithm to deep features extracted from the VGG-16 CNN, fine-tuned on the frontal view of the vehicles, followed by an SVM classifier.

Our approach in this paper is similar to previous studies on fine-grained classification. Deep neural network (DNN) based deep learning (DL) techniques have demonstrated state-of-the-art results in VMM classification. Their ability to select features, transform, and classify data within a single framework, in particular, draws practitioners looking for ready-to-use solutions from raw data [67]. However, in severe data limitations or the absence of relevant transfer learning problems, DNN-based DL’s advantages are drastically reduced [68]. We have proposed a hybrid CNN model fine-tuned on view-independent vehicle make and model datasets [5,69]. These datasets have a limited number of samples per class. The proposed model extracts deep features through the FC layer of a fine-tuned CNN and produces the features that best describe a vehicle for fine-grained vehicle classification using the Fisher discriminative least squares regression (FDLSR) module [3]. It then trains a linear classifier on these discriminative features and makes predictions. Compared to a fine-tuned CNN, the proposed hybrid model improves recognition accuracy by 2.1%. The improved accuracy shows that the hybrid CNN model is more tolerant to view-independent, small-scale vehicle datasets than pure DNN-based DL models. CNNs undoubtedly demonstrate superior classification performance in VMMR systems. Previous approaches used auxiliary networks in CNNs, altered CNN architectures, and introduced different loss functions to CNNs for fine-grained vehicle classification. Specific methodologies that worked directly on CNN feature maps to improve their generalization ability also improved classification results. However, we observe that the advantages of DNN-based DL are drastically reduced in cases of severe data limitations or the absence of a relevant problem for transfer learning [68]. To address this problem and utilize CNN’s ability to learn fine-grained features, we have proposed a hybrid CNN model fine-tuned on view-independent vehicle make and model datasets [5,69]. These datasets have a limited number of samples per class. The proposed model extracts deep features through an FC layer of a fine-tuned CNN and selects the most descriptive features using FDLSR. These transformed features exhibit improved inter-class disparity and intra-class similarity and are robust enough to be classified with a linear classifier. Table 1 lists some notable works in fine-grained image classification, especially VMMR.

## 3. Proposed Methodology

**Figure 1:**
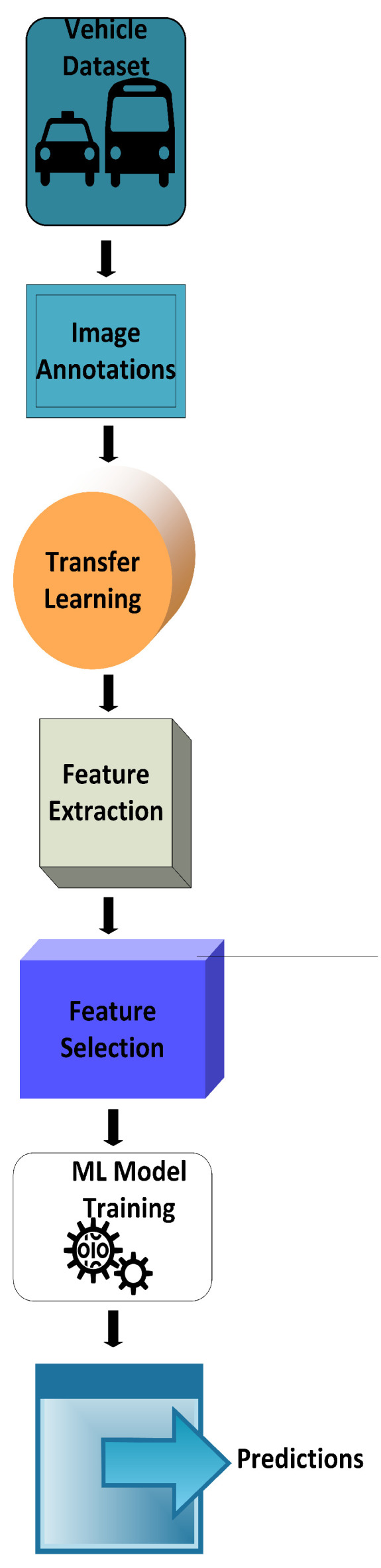
Proposed vehicle make and model recognition system.

In this section, we describe our proposed methodology in detail. Figure 1 provides an overview of our technique, and the subsequent sections describe each step in detail.

### 3.1. Transfer Learning on Fine-Grained Vehicle Datasets

**Figure 2:**
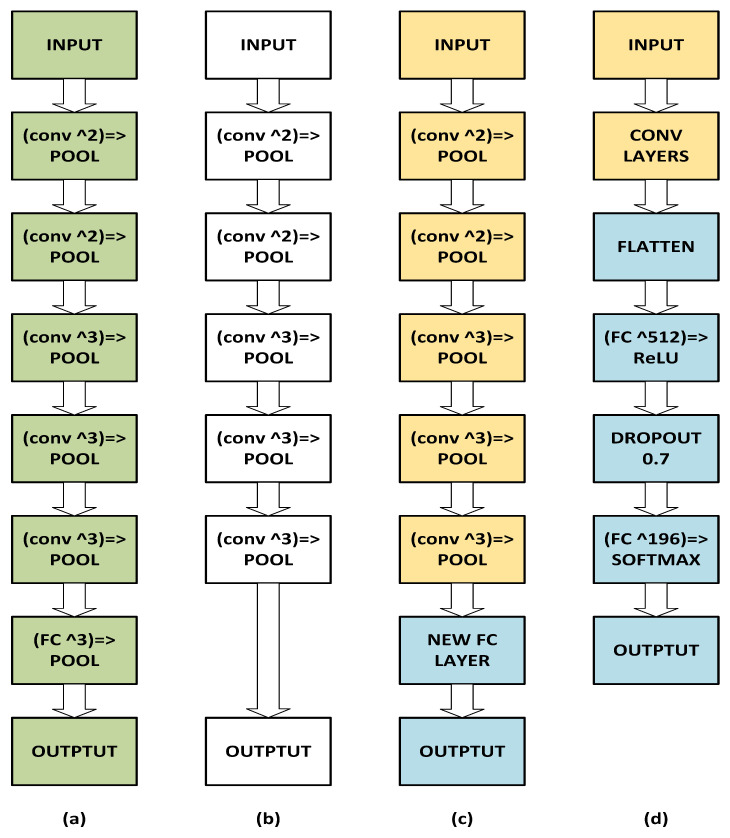
CNN architecture for transfer learning. (**a**) original CNN. (**b**) Our network without the FC layer. (**c**) Tuned network head without updating the base weights. (**d**) Fine-tuning of the FC layer head and the final CONV block.

Deep neural networks trained on large-scale datasets like ImageNet [73] and COCO [74] have shown remarkable transfer learning capabilities. We fine-tune pre-trained CNNs (VGG-16, ResNet-50, and ResNet-152) to extract class-specific, fine-grained features. On our training data, we applied data augmentation. Data augmentation is essential and always recommended for small datasets. Random rotations, zooms, and horizontal flips are among the parameters of a data augmentation object. To perform transfer learning with VGG-16, we load its architecture (with pre-trained ImageNet weights) from the disc and remove the fully connected layers. Figure 2a shows the original CNN. Figure 2b depicts our network without the FC layer. We then define a new fully connected layer head and freeze all VGG-16 CONV layers. At this point, training our model will only tune our network head and not update the base weights (Figure 2c). We reset our training and validation generators before unfreezing the final set of CONV layers, then unfreeze the final set of CONV layers. Figure 2d shows the final stage, which is to train our model to fine-tune the FC layer head and the final CONV block.

### 3.2. Feature Extraction with Deep Learning

The architecture of a pre-trained neural network allows us to use it as an arbitrary feature extractor. The input image propagates forward and stops at the pre-defined layer, allowing us to retrieve features from that layer. We can use powerful CNN features this way. We take our fine-tuned VGG-16 network and, similarly, allow an image to propagate forward to the dense layer (the first hidden layer of our fully connected layer) and extract features from it. This dense layer produces a 2048-dimensional feature vector. We can repeat the feature extraction process for each image in the dataset, yielding a total of N × 2048-dimensional feature vectors.

### 3.3. Feature Engineering with Fisher Discriminative Least Squares Regression (FDLSR)

To understand FDLSR [3], suppose we have a system QX=Y composed of a training dataset *X* with *m* features and *n* training examples. Let *Q* be the best-fit solution for the system such that QX≈Y. We use the optimization function of a least squares regression (LSR) model to find *Q*. The least squares regression (LSR) model finds the best possible solution by minimizing the residual sum of the squared (RSS) error [75]. The optimization function is written as:

(1) $$RSS=\sum {\left(y_{t}-\hat{y_{t}}\right)}^{2}.$$

However, solving a singular matrix for some RSS problems is difficult. Non-negative dragging values $\left\{\epsilon_{11},\epsilon_{12},\ldots ,\epsilon_{34}\right\}$ are added in the regularized RSS function under a technique called ϵ-dragging. The ϵ-dragging technique improves the inter-class margins, but it is observed that the class margins do not change significantly with each iteration, and DLSR does not consider the intra-class compactness of the relaxed labels. The Fisher criterion is applied to the ϵ-draggings to address this issue, increase inter-class separability, and improve intra-class compactness during each iteration. Thus, the Fisher discriminative least squares regression (FDLSR) [3] model can be formulated as a discriminative least squares regression (DLSR) model inspired by the Fisher criterion and ϵ-dragging method:

(2) $${min|QX-\left(Y+G.T\right)|}^{2}+\tau {\left|Q\right|}^{2}+\lambda Fisher\left(Y+G.T\right),$$

where

T>=0,

where *Q* is the projection matrix and *S* is the non-negative relaxation matrix. The matrix *Y* + *G* × *T* denotes the relaxed labels learned by the ϵ-dragging method. The first term is used to learn discriminative projection *Q* with relaxed regression labels, as shown in Equation (2). The third term aims to regularize the learned labels using the Fisher criterion. We introduce a transition variable *H* and rewrite our FDLSR model to understand better and optimize the Fisher function:

(3) $${min|QX-H|}^{2}{+\beta |H-\left(Y+G.T\right)|}^{2}+\tau {\left|Q\right|}^{2}+\lambda Fisher\left(H\right)$$

(4) $$Fisher\left(H\right)=\sum (|H-P{|}^{2}+|P_{m}-P{|}^{2}+|H{|}^{2}),$$

where

β,λ,τ>0

are scalars that weigh the corresponding terms in Equation (3), where *P* represents the relaxed labels of the *m*th class. *P* consists of *N* identical columns equal to the mean vector of all columns in *H*. *P* includes *n* identical columns equal to the mean vector of all columns in *H*.

To enhance intra-class compactness and inter-class separability of extracted features, we engineer the extracted features with the help of a Fisher discriminative least squares function in Equation (4). The extracted deep features *X* and their corresponding labels *Y* are loaded. These features are normalized, and their labels are converted into a one-hot encoded matrix. The FDLSR function uses a feature matrix (*X*), a label matrix (*Y*), and parameters β, τ, and λ as input to formulate the projection matrix *Q*. The FDLSR function undergoes 30 iterations to find a convergent solution. The function updates the transition *H*, projection *Q*, and relaxation *T* matrix during each iteration. The FDLSR algorithm projects the training data into a lower-dimensional subspace of *Q* by taking its dot product with the projection matrix. The transformed training set is now of the size *R* (c×n), where *c* represents the number of classes in a dataset. The pseudocode of FDLSR is showcased in Algorithm 1.
**Algorithm 1** Fisher Discriminative Least Squares Regression (FDLSR).** Initialization:** $\;Q=YX^{T}{\left(XX^{T}+\tau I\right)}^{{-}^{1}};T=0^{c\times n};H=Y;$
 $G=2Y-1^{c\times n};\hat{P}=\left[P_{1},P_{2},P_{3},...,P_{c}\right]$ Let $i=1;Q_{xy}=Q$ **while** $\;i<iterations_{max}$ **do**  $H=\frac{QX+\beta \left(Y+G\cdot T\right)-\lambda P-2\lambda \hat{P}}{1+\beta +2\lambda }$  $Q=HX^{T}{\left(XX^{T}+\tau I\right)}^{-1}$  T=max(G·(H−Y),0)  **if** ${∥Q-Q_{xy}∥}_{F}^{2}<10^{-4}$ **then**   **Stop**  **end if**  $i=i+1,Q_{xy}=Q$ **end while** **Output:** *Q*

### 3.4. Feature Classification Using Linear Classifier

We assume that features extracted by a fine-tuned CNN model are already robust and discriminative, as CNN can learn non-linear features. Therefore, once we have these transformed features, we can train off-the-shelf machine learning models such as Linear SVM and KNN on these features to recognize a new set of images. Support vector machines (SVMs) [4] are the supervised machine learning algorithms for classification and regression problems. For linearly separable cases, the optimization function is:

(5) $$y_{i}\left(mx_{i}+c\right)-1>=0$$

$$s.t.min\left\{1/2|w{|}^{2}\right\}.$$

For multiclass classification, n(n−1)/2 classifiers are trained in one-vs-one approach to classify samples from every pair of classes. The k-nearest neighbor algorithm considers the dimensions of the data points in a given space. It randomly selects data points from each class as class centers and calculates the distance between other samples and these center points. The commonly used metric to find the distance in a KNN algorithm is the Euclidean distance, which is given by:

(6) $$d\left(x_{1},x_{2}\right)=\sqrt{\sum (x_{1}-x_{2})}.$$

### 3.5. Overview of Proposed Algorithm

To conclude this section, we list the steps to implement our proposed algorithm.

**Step 1:**  Load the dataset.
**Step 2:**  Image preprocessing (annotations, augmentation).
**Step 3:**  Fine-tune the most suitable CNN model pre-trained on the ImageNet dataset.
**Step 4:**  Extract features from the fine-tuned CNN model’s fully connected (FC) layer. (The FC layer first flattens the feature map and gives it a vector form. The fully connected layer receives input from the last pooling or convolutional layer. The number of channels in the output feature maps extracted from a pre-trained VGG-16 is fixed at 512 and that of ResNet50 or ResNet152 at 2048).
**Step 5:**  Feature normalization.(Given the fixed size of the feature vector, this would produce 37,689 2048-dimension feature vectors and 8144 2048-dimension vectors for BoxCar21k and Stanford Cars, respectively.)
**Step 6:**  Begin with an 80/20 training validation split for both datasets. (Both are small and increasing the validation set might overfit the CNN model.)
**Step 7:**  Transform the features with FDLSR as described in detail in Section 3.3.
**Step 8:**  Feature normalization. (After applying the Fisher discriminative least squares function, the feature vectors are dimensionally reduced, yielding 2048 × 87-dimensional vectors and 2048 × 196-dimensional vectors for BoxCar21k and Stanford Cars, respectively.)
**Step 9:**  Train an off-the-shelf classifier. (e.g., SVM or KNN.)
**Step 10:**  Predictions.

## 4. Experimental Results and Discussions

### 4.1. Datasets

We have chosen the Stanford Cars dataset [5] and BoxCars21k [47] for our research. We chose the Stanford Cars dataset for its many classes and a few instances in each class. It is one of the earliest benchmark datasets. The dataset contains 16,185 view-independent images belonging to 196 classes of cars. The data are split nearly 50/50, with 8144 training images and 8041 testing images. Classes are at the level of make, model, and year.

**Figure 3:**
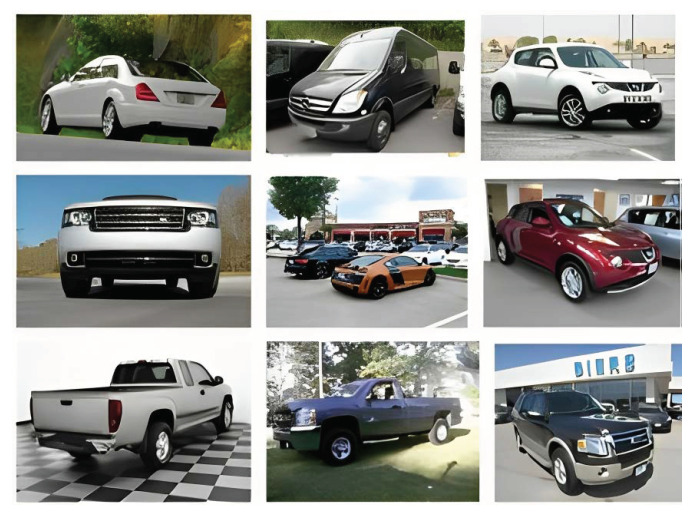
Images from the Stanford Cars dataset.

**Figure 4:**
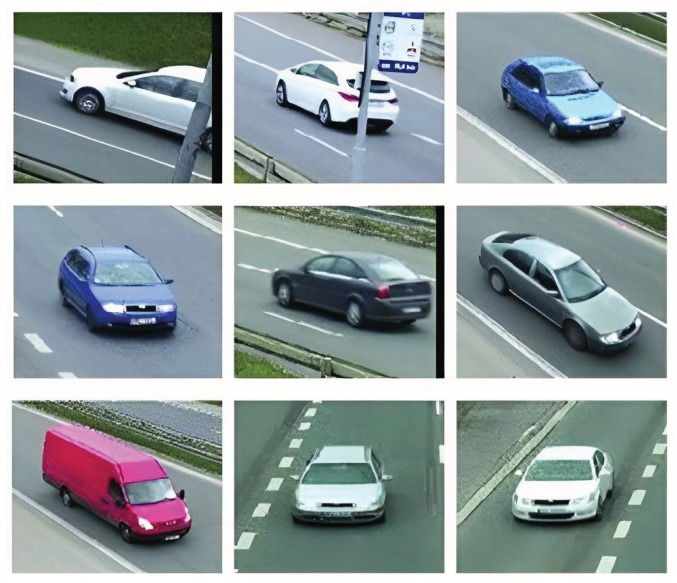
Images from the BoxCars21k dataset.

Figure 3 shows some images from the dataset. The sample images show the dataset’s view-independent nature and different illumination conditions. The BoxCars21k dataset contains 63,750 vehicle images of 148 fine-grained classes (make, model, and model year). Based on the fine categorization of the make-model hierarchy, the dataset is divided into easy, hard, and medium subsets. There is a considerable variation in viewpoints in the dataset. The dataset provides a 3D bounding box for each image. We have worked on the hard split, containing 37,689 images for training and 18,939 for testing, belonging to 87 fine-grained classes. Figure 4 shows sample images from the dataset.

**Figure 5:**
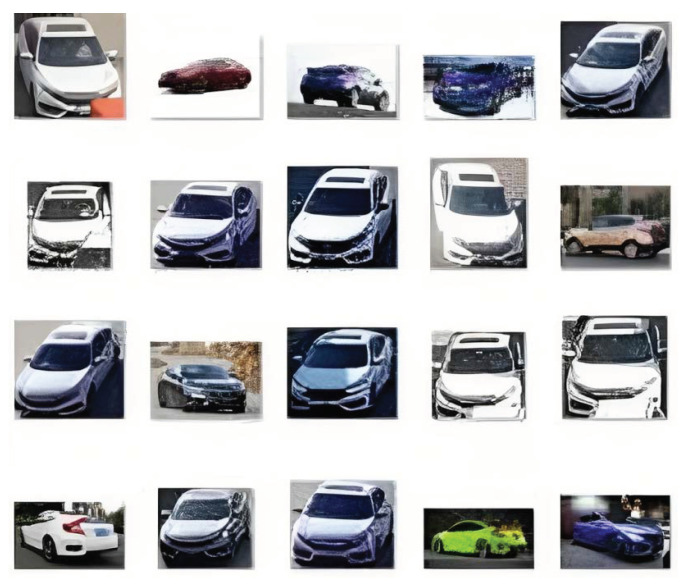
Images from the Pakistani cars dataset.

While carrying out experimentation for the choice of the best CNN model for feature extraction, another dataset was also used. Despite the ongoing research involving car make and model analysis, there is an absence of diverse datasets involving traffic dynamics in developing countries. Thus, we collected a comprehensive dataset that shall serve as a benchmark to further the research on traffic analytics to propose guidelines for ITS in developing countries like Pakistan. There are 129,000 images belonging to 94 different classes of vehicles on Pakistani roads to date. The dataset contains occluded images and partial and overhead camera views under low illumination. Images are labeled according to make, model, and generation; for example, HondaCity5 means Honda City 5th generation. Some examples are shown in Figure 5. Table 2 lists the main attributes of the datasets used for our experiments.

### 4.2. Choice of CNN

Considering the relatively small size of our datasets, training a deep neural network (DNN) can easily lead to overfitting. In such a situation, transfer learning is the natural solution. Transfer learning can achieve better performance with a relatively small dataset. In our proposed system, we trained the following popular CNN models to choose the best-performing model for our proposed approach.

- ResNet50 [69]
- ResNet152 [69]
- VGG-16 [76]
- InceptionV3 [77]
- MobileNet [78]

The dataset contains images taken by different users, imaging devices, and multiple view angles, ensuring numerous variations. As a result, the cars are not well-aligned, and some images have irrelevant backgrounds. The data were gathered by collecting and cleaning images from the internet and then cropping and cleaning images from Pakistani overhead traffic videos taken at different locations. Pictures taken from the internet are automatically annotated using the title and description the sellers had provided for each post. Figure 4 shows some images of the Honda Civic 10th generation from the dataset.

Most of these models are trained on the ImageNet dataset [73], which makes these CNN models ideal candidates for transfer learning. Each chosen model has its advantages. ResNet models, being most famous for transfer learning, help tackle the vanishing gradient problem and increase the training speed. They provide higher accuracy, especially for classification problems. These models learn the difference among the already learned features. If the learned feature is not helpful, then the final decision weights are set to zero for that particular feature. The main strength of the VGG models is that they are easy to understand and explain. They are suitable for typical two-class problems like cats vs. dogs classification. InceptionV3 has many advantages, as it reduces computational cost. It trains faster than the VGG family. The size of the model is smaller than VGG. MobileNet offers several advantages over other state-of-the-art convolutional neural networks, including reduced network size, reduced number of parameters, and faster performance, and it is helpful for mobile applications. Even though MobileNet has the advantage of smaller size, fewer parameters, and fast performance, it is less accurate than other state-of-the-art networks. Table 3 lists the test accuracies achieved by our chosen models for Stanford Cars and the local Pakistani on-road cars dataset.

### 4.3. Experimental Environment

All the experiments were performed on a GPU virtual machine with 16 GB RAM and a dual core CPU. Python 3.7 was used as the programming language.

### 4.4. Implementation Details

The most important thing to note is that the number of channels in the output feature maps extracted from a pre-trained VGG-16 (ResNet50, ResNet152) is fixed at 512 (2048). We fine-tune the pre-trained models with our proposed loss function to explore the pre-trained rich discriminative features of the VGG-16 (ResNet50, ResNet152) learned on a large ImageNet dataset. With the fixed size of the feature vector, this would produce 37,689 feature vectors of 2048 dimensions and 8144 vectors of 2048 dimensions for BoxCar21k (with 87 classes) and Stanford Cars (with 196 classes), respectively. After applying the Fisher discriminative least squares regression (FDLSR) function, the feature vectors are dimensionally reduced, yielding 2048 × 87-dimensional vectors and 2048 × 196-dimensional vectors for BoxCar21k and Stanford Cars, respectively.

To compare our approach with other state-of-the-art methods, we annotate and resize every image in the dataset to 224 × 224, then extract features using VGG-16 (ResNet50, ResNet152) pre-trained on ImageNet classification datasets. We began with an 80/20 training validation split for both datasets because both are small, and increasing the validation set might overfit the CNN model. We used stochastic gradient descent and batch normalization as regularizers. The learning rate of fully connected layers is kept at 0.0001, and we have trained no model for more than 100 epochs. Table 4 summarizes the hyperparameter values.

### 4.5. Evaluation Protocol and Measures

We conducted several experiments and analyzed our results to determine the best practices for vehicle make and model recognition using the chosen CNN architectures (VGG-16, ResNet50, and ResNet152). According to Table 3, these are the top three performing models. We have used top-1 and top-5 accuracy metrics to evaluate the performance of different fine-grained classification models. In fine-grained classification, differences between classes are pretty subtle, and the correct class is often in the top-k prediction, making top-k (k = 2, 3, 4, …) accuracy significantly higher than top-1 accuracy. We have exploited this accuracy gap to understand the performance of different classification models. We compared different classification models in this section in terms of accuracies, computational complexity, and other factors such as runtime.

#### 4.5.1. Test Accuracy Comparison

The different classification models trained on the same database with varying CNN model features have shown drastic variances in performance. Table 5 compares the accuracy of the FC layer, SVM, and our proposed classification model tested on the deep features of Stanford Cars. The highest top-1 test accuracy observed for the Stanford Cars database is 94.62% for our proposed model trained on fine-tuned ResNet152 features. The SVM model has performed better on ResNet50 features than other CNN features, with 94.44% accuracy, whereas the FC layer classification performance with ResNet152 features is comparably more convincing than others, with 90.37% top-1 accuracy. We observe that the accuracy gap between our proposed classifier’s top-1 and top-5 accuracy is minimal and ranges between 4–11%. This range stretches to 5–15% with SVM and 8–18% in the case of the FC layer. Additionally, this accuracy gap is associated with the final loss of the classifier, and with a higher gap, the losses are also higher. Since our proposed classifier has decreased this gap, minimal loss, i.e., 0.052, is observed by our classifier on ResNet152 features. The same trend is marked with the BoxCar21k database, as shown in Table 6.

#### 4.5.2. Computational Complexity Comparison

The complexity analysis of the FDLSR algorithm in Figure 2 is as follows [3]. When we update *T*, computation complexity is *O*(*ndc*). When updating *Q*, the complexity is

$$O(nd^{2}+d^{3}).$$

Therefore, the final computational complexity of updating *S* is

$$O(ndc+nd^{2}+d^{3})$$

Since the number of training samples and classes is much smaller than the dimensionality of the feature vector, the main time-consuming step is computing

$$X^{T}\cdot \left(1/\left(XX^{T}+\beta I\right)\right)$$

This term can be pre-computed because its value does not change during iteration. As a result, the final computational complexity of FDLSR [3] is

$$O(\left(nd^{2}+d^{3}\right)+2tndc),$$

where *t* is the number of iterations, *n* is the number of samples, *d* is the dimensionality of the data, and *c* is the number of classes in the dataset. The computational complexity of SVM is

$$O(nd^{2})$$

per iteration [4]. The proposed algorithm has the lowest computational complexity Since FDLSR converges in 30 iterations, while SVM takes 500 iterations to converge.

### 4.6. Runtime Comparison

**Figure 6:**
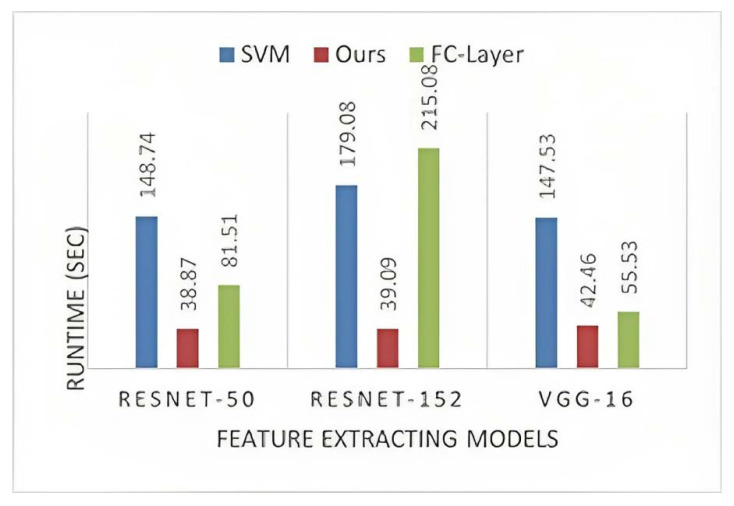
Runtime on the Stanford Cars dataset.

**Figure 7:**
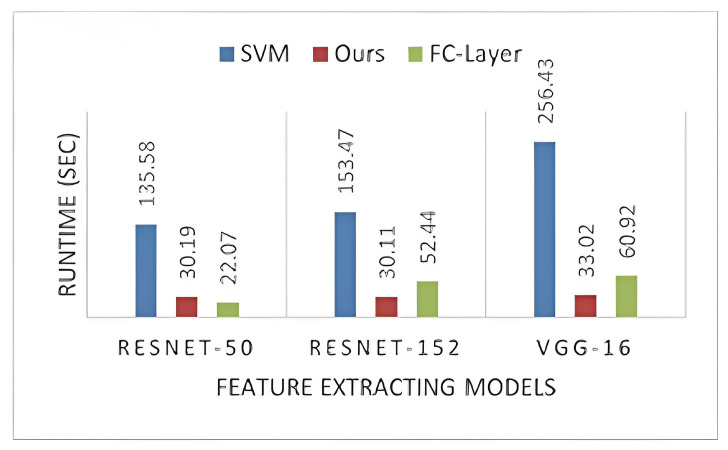
Runtime on the BoxCars21k dataset.

The extracted feature vector has the dimension 2048×N, where *N* is the number of sample images. Our proposed classifier has shown the lowest and almost equal runtime on both datasets (Figure 6 and Figure 7). Even with different CNN models, the runtime is constant, which shows that the number of sample images and the nature of extracted features have no impact on the runtime of our classification model. We can observe that the nature and the order of hidden layers in the FC layer affect its runtime. Similarly, SVM depends on the nature of the training set, as it has a varying runtime with different CNN model features.

### 4.7. Comparisons with State-of-the-Art Methods

Our proposed approach for VMMR presented in this paper outperforms several related VMMR works regarding classification accuracy. A comparison of our work with the results of other associated works on the Stanford Cars dataset is presented in Table 7. We have used three main categories of fine-grained recognition methods to draw comparisons. The first category is based on the attention mechanism, which includes a fully convolutional attention network (FCAN) [81], recurrent attention CNN (RA-CNN) [56], multi-attention convolutional neural network (MA-CNN) [82], dynamic time recurrent attention model (DT-RAM) [83], and trilinear attention sampling network (TA-SN) [84]. The second category, which is high-dimensional feature coding, includes a bilinear convolutional neural network (BCNN) [85], kernel pooling (KP) [86], higher-order integration of hierarchical convolutional activations (HIHCA) [87], boosted convolutional neural network (Boost-CNN) [88], HBP, and HBP with aggregated slack mask (HBPASM) [89]. Moreover, the third category is based on vehicle-specific characteristics, which include dual cross-entropy loss (DCEL) [90] and the global topology constraint network (GTCN) [91]. Using the ResNet152 model as the 379 feature extractor, the proposed fine-grained classification model achieves the best accuracy of 94.61% on the Stanford Cars dataset.

## 5. Conclusions

This paper proposed a novel classifier based on FDLSR to solve the problem of view-independent car make and model classification. For our research, we have chosen the Stanford Cars dataset and BoxCars21k. The former was selected for its large number of classes and a small number of instances in each class, while the latter was selected for the considerable variation in viewpoints in the dataset. We also introduced a Pakistani cars dataset and conducted experiments for CNN selection on it. Preexisting CNN models were considered for feature extraction and after extensive experimentation, ResNet-50, ResNet-152, and VGG-16 were selected. Selected features were fed to our proposed classifier. Experimental results show that our proposed classifier achieves substantially better results than the existing state-of-the-art approaches. Our method deals with the main problem deep neural networks face, i.e., poor performance on a small training set. Due to FDLSR’s ability to increase inter-class distance and decrease intra-class distances, class boundaries become more defined. We see superior performance on datasets with a large number of classes and with a small number of samples per class. Our proposed classifier has the shortest run time independent of the type of features fed to the classifier. For future work, we plan to conduct experiments on the Pakistani cars dataset and implement incremental learning for feature extraction.

## Figures and Tables

**Table 1:** Summary of some Notable Works.

Year & Author	Objective	Dataset	Methodology	Result	Remarks
Biglari, M., 2018 [49]	To design a novel cascaded part-based system for VMMR	CompCars BVMMR	Novel greedy parts localization, and a practical multi-class data mining algorithm to detect discriminative vehicle region. Use of cascaded scheme to speed up the mechanism	up to 80% speed optimization. 97.01% accuracy on CompCars	Cascaded system performs with higher speed and accuracy than the baseline system.
Manzoor, M. A., 2019 [44]	To present a unique and robust real-time VMMR system which can handle unique set of challenges	NTOU-MMR	Used Histogram of Oriented Gradient (HOG) and GIST to represent the images and SVM and RF to classify the vehicles	97.20% with GIST features and SVM	System is well-suited for situations where vehicles are partially occluded, partially out of the image frame or poorly visible due to low lighting.
Benavides, N., 2019 [70]	Fine-tuning of a pre-trained CNN on a VMMR dataset	Stanford Cars	Transfer learning and fine-tuning of VGG16 Use of dropout, data augmentation and downsizing of dense layer	96.3% Top-5 Test Accuracy	Techniques used for dimensionality reduction are found crucial in fine-grained vehicle classification
Ma, Z., 2019 [59]	Improve generalization ability of CNNs for fine-grained classification	Stanford Cars, CompCars	Inserting Channel Max Pooling Layer (CMP) between the fully connected layers and the convolutional layer	97.89% by DenseNet161 on CompCar	CMP improves the performance of a network. It reduces the number of parameters in a neural network
Anwar, S., 2020 [71]	Comparison of general CNN classifiers and fine-grained classifiers	Stanford Cars, FGVC Aircrafts, Flowers, NA Birds	Transfer learning and fine-tuning of CNNs on FGVC datasets	94.5% by DenseNet161 on Stanford Cars	Traditional CNNs outperformed fine-grained classifiers in FGVC mainly because traditional CNNs are pre-trained CNNs.
Chang, D., 2020 [65]	Obtain fine-grained features using single-loss function	Birds, FGVC Aircraft, Flowers102, Stanford Car	Applied mutual-channel loss (MC-loss) directly to the feature channels	94.1% with ResNet-50 features on Stanford Cars	MC-Loss does not need fine-grained bounding-box. It can be applied to any network architecture. Does not need any extra parameter for tuning
Naseer, S., 2020 [66]	Proposal of a VMMR framework	NTOU MMR	Fine-tuning of VGG-16. Deep features extraction dimensionality reduction by GA. Classification using SVM.	98.20%	SVM performs better than any other classifier on fine-grained features and results are comparable to the state-of-the-art methods
Boukerche, A., 2021 [72]	LRAU to enhance the feature extraction ability of CNN architectures for VMMR.	Stanford Cars, CompCars, NTOU-MMR	Proposed LRAU extracts the discriminative part features by generating attention masks to locate the key points of a vehicle	93.94% on Stanford Cars	Model achieves excellent fine-grained recognition performance and can be used in a real-time environment

**Table 2:** Main attributes of datasets used in experiments.

Dataset	Year	Samples	Diversity	Annotations	Image Resolution	No. of Classes	Train/Test Split
BoxCars21k [47]	2016	63,750	View-Independent	Make, model year	Low	148	70/30
Stanford Cars [5]	2013	16,185	View-Independent	Make, model year	Mixed	196	50/50
Pakistani Cars	2022	129,000	View-Independent	Make, model generation	Low	94	60/40

**Table 3:** Accuracies achieved.

No.	Model Name	Test Accuracies (Pakistani Cars)	Test Accuracies (Stanford Cars)
1	ResNet50	90%	92%
2	ResNet152	90%	82%
3	VGG-16	70.80%	71%
4	Inception V3	70%	58%
5	MobileNet		

**Table 4:** Summary of hyperparameters.

Hyperparameters	Value	Rationale
Optimizer	SGD	Recommended for ResNet models [79]
Learning Rate	0.0001	Recommended for ResNet models [80] because we do not want to change too much what is previously learned
Batch Size	64	Best accuracy
Epoch	<100	Avoid overfitting

**Table 5:** Stanford Cars test accuracies.

Classification Models	Feature Extracting Models	Top-1 Accuracy	Top-5 Accuracy	Final Loss
FC Layer	ResNet50	90.37	98.40	0.096
	ResNet152	90.52	98.15	0.094
	VGG-16	76.32	94.47	0.236
SVM	ResNet50	94.44	99.02	0.056
	ResNet152	94.17	99.03	0.053
	VGG-16	81.76	96.40	0.183
Ours	ResNet50	94.16	98.98	0.053
	ResNet152	94.62	99.09	0.052
	VGG-16	86.17	97.51	0.166

**Table 6:** BoxCars21k test accuracies.

Classification Models	Feature Extracting Models	Top-1 Accuracy	Top-5 Accuracy	Final Loss
FC Layer	ResNet50	96.15	99.56	0.038
	ResNet152	97.91	99.92	0.020
	VGG-16	93.91	99.25	0.060
SVM	ResNet50	98.40	99.99	0.016
	ResNet152	98.41	99.99	0.019
	VGG-16	93.21	99.44	0.082
Ours	ResNet50	98.74	100	0.013
	ResNet152	98.88	100	0.012
	VGG-16	96.84	100	0.031

**Table 7:** Comparison of the Proposed Method with State-of-the-art Methods.

Method	Accuracy %
Channel Max Pooling (CMP) [59]	93.71
Spatially weighted pooling (SWP) [58]	93.1
Mutual-channel loss (MC) [65]	90.85
Recurrent-attention CNN (RA-CNN) [56]	92.5
Multi attention CNN (MA-CNN) [82]	92.8
Fully convolutional attention network (FCAN) [81]	89.1
Dynamic time recurrent attention model (DT-RAM) [83]	93.1
Trilinear attention sampling network (TA-SN) [84]	93.8
Kernel pooling (KP) [86]	92.4
Higher-order integration of hierarchical convolutional activations (HIHCA) [87]	91.7
Bilinear convolutional neural network (BCNN) [85]	92.1
Dual cross-entropy loss (DCEL) [90]	93.3
Global topology constraint network (GTCN) [91]	94.3
Our proposed method	94.61

## Data Availability

This research used publicly available datasets for experimentation and analysis purposes.
